# Effects of Visual Deprivation on Remodeling of Nodes of Ranvier in Optic Nerve

**DOI:** 10.1523/ENEURO.0194-22.2022

**Published:** 2022-11-04

**Authors:** Erin N. Santos, William C. Huffman, R. Douglas Fields

**Affiliations:** Section on Nervous System Development and Plasticity, *Eunice Kennedy Shriver* National Institute of Child Health and Human Development, National Institutes of Health, Bethesda, Maryland 20892

**Keywords:** astrocyte, binocular visual deprivation, dark rearing, myelin, node of Ranvier, sensory deprivation

## Abstract

Oligodendrocytes, the myelinating cells of the CNS, promote rapid action potential conduction along axons. Changes in the geometry of gaps between myelin segments, known as nodes of Ranvier, affect the conduction speed of neuronal impulses and can ultimately alter neural synchronization and circuit function. In contrast to synaptic plasticity, much less is known about how neural activity may affect node of Ranvier structure. Recently, perinodal astrocytes have been shown to remodel nodes of Ranvier by regulating thrombin proteolysis, but it is not known whether neural activity influences this process. To test this hypothesis, we used transgenic mice with astrocytic expression of a dominant-negative vesicle-associated membrane protein 2 ([gfap]dnVAMP2) to reduce exocytosis of thrombin inhibitors, modulating astrocytic regulation of paranodal loop attachment to induce nodal remodeling, under normal conditions and in adult mice maintained in darkness from postnatal day 40 (P40) to P70. This mechanism of nodal lengthening proceeded normally following binocular visual deprivation (BVD). The effect of BVD on nodal plasticity in animals with unimpaired astrocyte function has not been previously investigated. We find that when exocytosis from astrocytes was unimpaired, nodal gap length was not altered by BVD in adult mice. We conclude that if perinodal astrocytes participate in activity-dependent myelin remodeling through exocytosis, then, as with synaptic plasticity in the visual system, the process must be driven by alterations in neuronal firing other than those produced by BVD.

## Significance Statement

Recent studies show that nodes of Ranvier can undergo activity-dependent remodeling, but the mechanisms are unclear. Indeed, whether sensory deprivation alters nodes of Ranvier has not been tested. An implicit assumption in studies of sensory experience in synaptic plasticity (e.g., the classical Hubel and Wiesel studies and others) is that binocular visual deprivation (BVD) does not change myelin to alter action potential conduction. Here, we test that assumption and also determine the effect of BVD on a specific astrocyte-mediated mechanism of node of Ranvier plasticity in optic nerve of adult mice. The astrocyte-mediated nodal gap lengthening proceeded normally in adult mice maintained in the dark for 30 d. Second, the absence of plasticity of nodes of Ranvier in controls parallels our understanding of synaptic plasticity in visual cortex following BVD.

## Introduction

Nodes of Ranvier are the small gaps along myelinated axons where voltage-activated sodium channels in the axolemma generate action potentials to increase impulse conduction velocity by saltatory conduction. Even slight changes in the structure of nodes of Ranvier can alter conduction velocity and influence spike arrival time and neural circuit function (Dutta et al., 2018). Therefore, if nodal gap remodeling is regulated by sensory experience, nodal plasticity could be a form of experience-dependent plasticity complementing synaptic plasticity.

Studies in the visual system using binocular and monocular visual deprivation have provided our fundamental understanding of how synaptic plasticity is regulated by sensory experience (Wiesel and Hubel, 1965; for review, see Hooks and Chen, 2020). It was assumed in these classical studies, and others, that node of Ranvier structure, and thus conduction velocity through the optic nerve, was not altered by visual experience, but recent studies show that the structure of myelin and the node of Ranvier can change in response to action potential activity (Fu et al., 2009; Tagoe et al., 2014; Bellesi et al., 2018; de Vivo and Bellesi, 2019; Benamer et al., 2020; Cullen et al., 2021). It is important, therefore, to determine how this new form of myelin plasticity may be influenced under similar conditions used in studies of visual system synaptic plasticity. If nodal structure and conduction velocity in optic nerve are altered by visual experience, thus affecting the synchrony of spike time arrival in the lateral geniculate nucleus and visual cortex, this could augment, counteract, or even drive some of the outcomes of previous research on synaptic plasticity performed before the recent discovery of myelin plasticity. It is unclear the extent to which nodal gap lengthening may be altered by sensory deprivation, particularly after mature myelin has formed. Here we test whether prolonged binocular visual deprivation (BVD) by maintaining adult mice in total darkness for 30 d alters the length of nodes of Ranvier in optic nerve.

Second, current studies of nodal plasticity in adult animals are observational, leaving the cellular and molecular mechanisms unclear. Identifying these mechanisms is particularly important in the context of new research showing plasticity of myelin during learning in adult animals (McKenzie et al., 2014; Kato et al., 2020; Pan et al., 2020; Steadman et al., 2020). To advance the field, it is essential to begin to investigate specific mechanisms of node of Ranvier plasticity and to determine whether they are influenced by action potential activity. Therefore, we tested a specific mechanism known to modulate nodal gap length in mature compact myelin to determine whether it is influenced by functional activity in axons. This plasticity is driven by a transgene, and the effects of neural activity on this mechanism of nodal plasticity in adult mice were investigated by maintaining the animals in the dark for 30 d.

In this mechanism of myelin plasticity, perinodal astrocytes can modify action potential conduction velocity by regulating the structure of nodes of Ranvier and the thickness of the myelin sheath via vesicular release of thrombin protease inhibitors (Dutta et al., 2018). If uninhibited, thrombin activity severs the attachment of paranodal loops of myelin from the axon via cleavage of the axoglial cell adhesion molecule NF155 (Dutta and Fields, 2021). In transgenic mice with the astrocyte-specific human glial fibrillary acidic protein (GFAP) promoter driving expression of a dominant-negative (dn) fragment of the vesicular release protein ([gfap]dnVAMP2), astrocyte exocytosis of thrombin inhibitors is reduced by ∼50% (Dutta et al., 2018). Expression of [gfap]dnVAMP2 results in lengthening of the nodal gaps in optic nerve, as the paranodal loops adjacent to the perinodal astrocyte detach from the axon, and in thinning of the myelin sheath, as the outer layer of myelin, associated with the detached paranodal loops, becomes resorbed into the oligodendrocyte (Fields and Dutta, 2019). Although the change in nodal gap length induced by the astrocyte-mediated mechanism is less than a micrometer, it represents an ∼30% increase in nodal gap length and causes an ∼20% slowing of conduction velocity in optic nerve (Dutta et al., 2018). The slower conduction through the optic nerve results in 6.7 ms increased latency of spike time arrival in visual cortex, which is a substantial delay from the mean 72.8 ms latency to peak visually evoked potential in the absence of nodal gap lengthening and myelin sheath thinning produced by this mechanism. The decreased speed of impulse conduction reduced visual acuity by 0.01 cycles/°. All of these effects are statistically significant and reversed by administering doxycycline (DOX) to terminate the expression of the transgene or by using thrombin inhibitors.

We chose to explore the effect of BVD on this astrocyte-regulated mechanism because there is a clear process by which activity could regulate lengthening of the nodal gap via this astrocyte-driven mechanism in adult animals. Activity-dependent signaling between axons and astrocytes is well established (Orkand et al., 1966), and action potentials in axons can cause a rise in astrocytic intracellular calcium (Kriegler and Chiu, 1993; Hamilton et al., 2008; Fields and Ni, 2010). Since astrocyte exocytosis is sensitive to changes in intracellular calcium concentration, modulation of astrocyte-regulated myelin remodeling by action potential firing is feasible. This possibility was tested using [gfap]dnVAMP2-expressing mice maintained under normal visual conditions or BVD and comparing the extent of nodal remodeling that occurs under each condition. If the astrocyte-mediated nodal gap lengthening is responsive to BVD, decreased functional activity would be predicted to further reduce exocytosis of thrombin inhibitors from astrocytes and result in longer nodes of Ranvier in [gfap]dnVAMP2-expressing mice after BVD by maintaining adult mice in the dark for 30 d, compared with these mice in normal visual conditions.

Alternatively, many other cellular mechanisms may be responsible for the activity-dependent changes in nodal gap length reported previously, including expansion or contraction of the axon at the node (Ford et al., 2015) or oligodendroglia deposition and removal of myelin. If so, sensory deprivation could alter nodal gap length although the astrocyte-driven mechanism is inhibited by *dnVAMP2* transgene expression.

The results indicate that BVD by maintaining adult mice in darkness for 30 d did not influence the perinodal astrocyte-dependent nodal lengthening, and did not alter nodal gap length in mice in which exocytosis in astrocytes was unimpaired. Therefore, if the astrocyte-mediated mechanism of nodal gap lengthening is regulated by neural impulse activity, BVD is not sufficient to drive the process. This is consistent with conclusions from a large body of literature on the effects of visual deprivation on synaptic plasticity in the visual system.

## Materials and Methods

### Transgenic mouse production and maintenance

We used a line of transgenic *[gfap]dnVAMP2* mice of both sexes, in which there is astrocyte-specific expression of a DOX-regulated VAMP2 cytoplasmic fragment containing amino acids 1–96, which acts as a dominant-negative inhibitor of interactions between vesicular SNARE-containing vesicles and target SNAREs, such as Snap23. Astrocyte-specific *GFAP* promoter drives the expression of the “tet-OFF” tetracycline (tet) transactivator GFAP.tTA. Another transgenic line expresses a tet operator (tetO)-regulated dnVAMP2 cytoplasmic fragment and enhanced green fluorescent protein (EGFP) and LacZ reporter genes *tetO.VAMP2*. Details of producing GFAP.tTA and *tetO.dnVAMP2* lines are as described previously (Pascual et al., 2005). This line of mice was developed and provided by Ken McCarthy (University of North Carolina, Chapel Hill, NC; Pascual et al., 2005). These lines were maintained in a heterozygous state and backcrossed onto a C57BL6/J genetic background (The Jackson Laboratory) for >20 generations.

**Figure 1.**
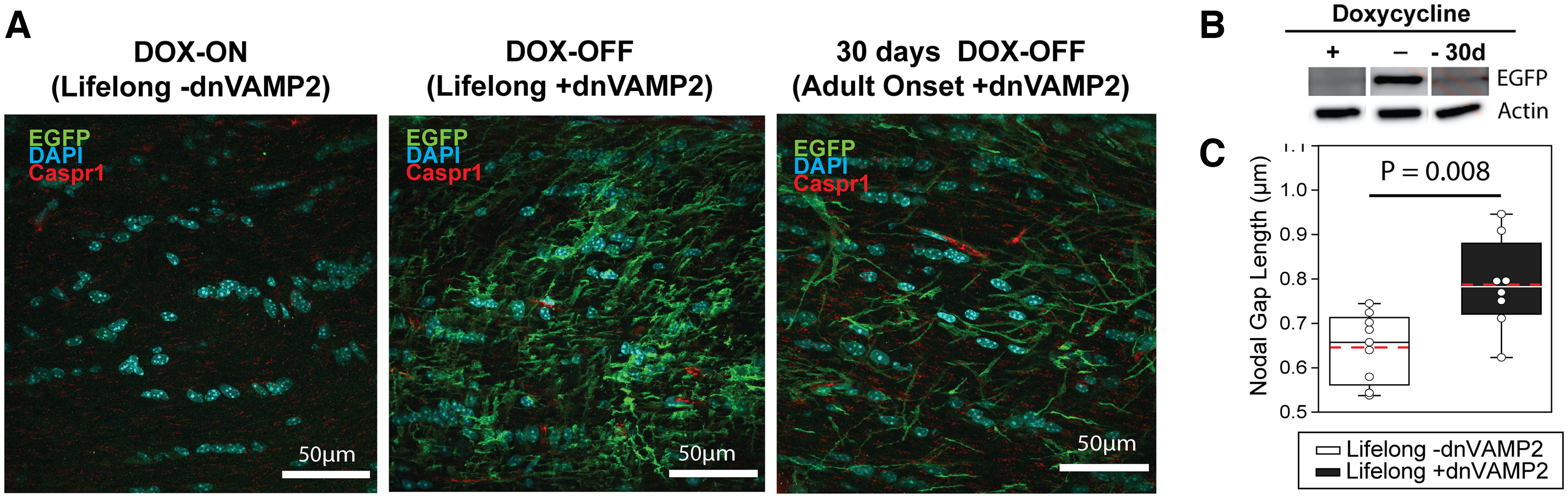
Doxycycline regulation of astrocyte-specific transgene expression to induce nodal remodeling. ***A***, EGFP expression serves as a marker for dnVAMP2 expression in the optic nerve. EGFP expression (green) was absent in the optic nerves of *[gfap]dnVAMP2* mice supplied DOX (DOX-ON) from gestation through adulthood (P70). In contrast, EGFP expression (green) was prevalent in astrocytes in the optic nerves of *[gfap]dnVAMP2* mice on a diet that did not contain DOX (DOX-OFF) from gestation through adulthood (P70). EGFP expression in astrocytes was also elevated when *[gfap]dnVAMP2* mice were raised on a DOX-ON diet through development, but DOX was removed from their diet from P40 to P70 (30 d DOX-OFF). The paranodal region is marked by Caspr1 labeling in red. ***B***, Western blotting for EGFP expression confirmed the results of immunocytochemistry. Actin was used as a loading control. ***C***, Box plots of average nodal gap length measured per animal. Individual points represent the average of 10 microscope fields per animal. Maximum, minimum, and median nodal gap lengths are represented within the box plot as whiskers (top and bottom) and mid-line (solid black). The mean nodal gap length of all animals within each condition is marked with a dashed red line. Optic nerve nodal gap length was larger in lifelong +dnVAMP2 mice than in lifelong –dnVAMP2 mice (mean nodal gap length ± SEM: 0.646 ± 0.026 vs 0.787 ± 0.036 μm; lifelong –dnVAMP2 12 h light/dark cycle vs lifelong +dnVAMP2 12 h light/dark cycle, respectively; *t* test, *t*_(12)_ = −3.17, *p* = 0.008).

Biogenic offspring of these two lines (*[gfap]dnVAMP2*) were maintained on 200 mg/kg DOX-supplemented food (Bio-Serv) to block transgene expression (–dnVAMP2) or fed a regular diet to allow transgene expression in astrocytes (+dnVAMP2; Fig. 1*A*). A previous study (Dutta et al., 2018) shows that both dnVAMP2 and EGFP tetO transgenes coinherit, implying that they are cointegrated on the same chromosome. The specificity and dynamics of DOX regulation of the EGFP reporter and dnVAMP2 gene have been published previously, including the time course of expression and the cessation of gene expression with DOX withdrawal, and the cellular specificity in astrocytic expression (Pascual et al., 2005; Halassa et al., 2009; Sardinha et al., 2017; Dutta et al., 2018). Studies have demonstrated a high degree of colocalization of EGFP with cytosolic dnSNARE in astrocyte cell culture (Halassa et al., 2009) and have shown that the amount of EGFP and dnVAMP2 mRNA transcripts are directly correlated and vary similarly between *[gfap]dnVAMP2* animals (Sardinha et al., 2017). This cointegration and consequent coinheritance means that the highly sensitive reporter gene EGFP can be reliably used as a surrogate reporter for the *dnVAMP2* transgene in our studies.

In lifelong –dnVAMP2 experiments, *[gfap]dnVAMP2* transgenic mice were maintained on a DOX-supplemented diet from gestation through adulthood. Similarly, in the lifelong +dnVAMP2 condition, regular feed was provided to *[gfap]dnVAMP2* mice from gestation through adulthood. In adult-onset experiments, *[gfap]dnVAMP2* transgenic mice were maintained on a DOX-supplemented diet through gestation and development, and DOX was removed from the diet at postnatal day 40 (P40) until the end of the experiment at P70 to induce transgene expression only during adulthood. Expression of EGFP reporter for dnVAMP2 has been shown to increase within a week of removing DOX from the diet (Dutta et al., 2018). All animals were killed by cervical dislocation at P70 so that optic nerves could be removed for nodal gap length quantification.

All mice were maintained in group housing under specific pathogen-free conditions with access to food and water *ad libitum* according to a protocol approved by the *Eunice Kennedy Shriver* National Institute of Child Health and Human Development (NICHD) Animal Care and Use Committee. All animal studies were approved by the NICHD Animal Care and Use Committee. Both sexes were used, but because of the limited production of transgenic animals, this study did not distinguish between the sexes of the animals.

### Binocular visual deprivation

All mice were raised under a 12 h light/dark cycle until P40, when mice are mature and myelin is fully formed. Mice placed in the control condition continued to be maintained under a 12 h light/dark cycle until P70. Mice in the BVD condition were moved into a separate room at P40 for 30 d (until P70) where they were maintained under 24 h darkness; all feeding and cage changes, as well as killing and tissue collection, occurred under a red light.

### Immunohistochemistry

Mice were deeply anesthetized with isoflurane and killed by cervical dislocation before eyes were removed and optic nerves were dissected. Optic nerves were fixed in 4% paraformaldehyde (Electron Microscopy Sciences) in PBS overnight at 4°C before being transferred to 30% sucrose in PBS for 3 d. Tissue was embedded in optimal cutting temperature embedding medium (O.C.T., Thermo Fisher Scientific), sectioned into 12-μm-thick slices, and mounted on glass slides. Embedded tissue and slides were stored at −20°C. Slides were rehydrated with PBS. A blocking solution of 5% normal goat serum (Jackson ImmunoResearch), 0.5% Triton X-100 (Sigma-Aldrich), and 0.5% bovine serum albumin (BSA; Sigma-Aldrich) was applied for 1 h. Primary antibodies were diluted in the blocking solution to the following concentrations: anti-contactin-associated protein 1 (Caspr1; 1:50; Mouse Monoclonal, catalog #K65/35, University of California, Davis/National Institutes of Health (NIH) NeuroMab Facility; RRID:AB_2877274); anti-Na_v_1.6 (SCN8A; 1:100; rabbit polyclonal, catalog #ASC-009, Alomone Labs; RRID:AB_2040202); and anti-GFP (1:1000; rabbit polyclonal, catalog #ab290, Abcam; RRID:AB_303395). Primary antibodies incubated overnight at 4°C. Slides were washed with PBS plus 0.5% Triton X-100 before secondary antibodies [goat anti-mouse IgG Alexa Fluor 633, catalog #A-21052, Thermo Fisher Scientific (RRID:AB_2535719); goat anti-rabbit IgG Alexa Fluor 568, catalog #A-11011, Thermo Fisher Scientific (RRID:AB_143157); goat anti-rabbit IgG Alexa Fluor 488, catalog #A-11034, Thermo Fisher Scientific (RRID:AB_2576217)] were applied for 2 h at room temperature at 1:1000 dilution in PBS, 0.5% Triton X-100, and 0.5% BSA. Slides were rinsed with PBS plus 0.5% Triton X-100 and PBS before being sealed with Prolong Diamond Antifade Mountant with DAPI (catalog #P36962, Thermo Fisher Scientific). Images were captured by confocal laser microscope (model Fluoview FV300, Olympus). To visualize EGFP expression in the optic nerve, 21 optical sections at 0.2 μm intervals were obtained with a 60× lens (1× zoom), and a single-plane maximum projection was formed. Samples from control and experimental animals were imaged with the same microscope settings.

### Western blots

Optic nerves from lifelong –dnVAMP2, lifelong +dnVAMP2, and adult-onset +dnVAMP2 mice were collected to confirm doxycycline regulation of the transgene. Optic nerves were lysed in M-PER (Mammalian Protein Extraction Reagent) plus 2× NuPage Sample Buffer with 5% β-mercaptoethanol and protease inhibitors (catalog #11836170001, Roche Diagnostics), and loaded for electrophoreses. Total protein was resolved by SDS-PAGE on 4–12% NuPAGE Bis-Tris gels (Thermo Fisher Scientific), transferred to PVDF membrane (ImmobilonP, Millipore), and blocked in TBS (10 mm Tris-Cl, pH 7.5, 0.9% NaCl) containing 0.1% (v/v) Triton X-100 (TBS-T) and 5% (w/v) BSA for 2 h at room temperature. β-Actin (catalog #8457, Cell Signaling Technology; RRID:AB_10950489) and EGFP (catalog #ab290, Abcam; RRID:AB_303395) primary antibodies were applied at a 1:2000 dilution in TBS-T and 5% BSA overnight at 4°C. Primary antibodies were visualized with HRP-conjugated secondary antibodies (1 ml; catalog #NA9340, Cytiva; RRID:AB_772191) at 1:4000 dilution and enhanced chemiluminescence.

### Nodal gap measurement

Images were captured by confocal laser microscopy (with 60× lens with 4× zoom; model Fluoview FV300, Olympus). Nodes of Ranvier were visualized by immunohistochemical staining of Caspr1, a cell adhesion molecule expressed in the paranodal region flanking the nodal gap and verified by immunohistochemistry for voltage-gated sodium channels (Na_v_1.6), which are concentrated at the nodes of Ranvier. A z-series of 10 optical sections at 0.2 μm intervals was taken and a single-plane maximum projection was formed. The lengths of nodes of Ranvier were measured using NIH ImageJ software. The length of each node was determined by measuring the distance between opposing Caspr1-labeled paranodes. In total, 25,747 nodes of Ranvier were measured in these studies, from 41 transgenic mice of both sexes. Nodal gap measurements were collected while blinded to the experimental condition.

### Statistics

For statistical analysis, all nodes of Ranvier in a microscope field (53 μm^2^) were measured to adequately sample the within-field variance and averaged. The mean value of all nodes (*n*) in each microscope field was calculated (average, 60 nodes/field), and the average nodal gap length for each animal (*N*) was determined by averaging 10 microscope fields per animal to avoid artificially inflating the sample size (Lazic et al., 2018). In this way, the difference in mean nodal length in the control and experimental groups were compared by a two-sample *t* test, with *N* being the number of animals used for statistical calculations.

Statistical analysis was performed using Minitab software (Minitab), applying a two-sample *t* test to compare the mean nodal gap length between visual conditions (12 h light/dark cycle vs 24 h darkness) within each genetic background condition or to compare the mean nodal gap length of animals across different genetic backgrounds. The mean (SEM), *t*-statistic test value, and degrees of freedom are reported in standard American Psychological Association format. Box plots were created using SigmaPlot 14.5 by displaying mean nodal gap lengths for each animal as individual data points. Maximum, minimum, and median nodal gap lengths are represented within the box plot as whiskers (top and bottom) and mid-line (solid black). The mean nodal gap length of all animals within each condition is marked with a dashed red line. Values of less than *p* = 0.05 were considered to be significantly different.

To analyze the sources of variance across all conditions in all of the experiments performed in this study, a two-way ANOVA using a general linear model fit was performed to determine the influence of the following two factors of interest: (1) vesicle release from astrocytes (DOX Condition); and (2) sensory deprivation (Visual Experience), on nodal gap length across all conditions, as well as the degree of interaction between these two factors. For this comprehensive analysis, all nodes of Ranvier in a microscope field were measured and the mean value of each field was taken for analysis. This analysis represents a sample size of 410 microscope fields from 41 animals. Box plots were created using SigmaPlot 14.5 by displaying mean nodal gap lengths for each field, and the outlying data points are shown. Maximum, minimum, and median nodal gap lengths are represented within the box plot as whiskers (top and bottom) and mid-line (solid black). The mean nodal gap length of all fields within each condition is marked with a dashed red line. Values of less than *p* = 0.05 were considered to be significantly different.

## Results

### Astrocyte-regulated node of Ranvier plasticity proceeds normally during long-term binocular visual deprivation

Vesicular release of thrombin inhibitors from perinodal astrocytes regulates the length of the nodal gap and thickness of the myelin sheath in adult animals (Dutta et al., 2018). To determine whether sensory deprivation influences this mechanism of nodal plasticity, transgenic mice were used that express dnVAMP2 specifically in astrocytes under the regulation of DOX. EGFP expression serves as a reporter, and gene expression is prevented by administering DOX (Fig. 1). In the absence of DOX, EGFP was expressed specifically in astrocytes as shown by immunohistochemistry (Fig. 1*A*). This gene expression was evident in mice lacking DOX treatment throughout life, including during gestation (lifelong +dnVAMP2), or when DOX was eliminated from the diet of adult mice for 30 d (adult-onset +dnVAMP2; Fig. 1*A*). Mice raised on a diet containing DOX to suppress the transgene, and then switched to a regular diet for 30 d during adulthood showed increased expression of EGFP. Immunoblot for EGFP expression confirmed that mice maintained on a diet containing DOX throughout life have no EGFP expression in optic nerve, but high transgene expression in optic nerves of [gfap]dnVAMP2 mice on a DOX-free diet was evident by immunoblot (Fig. 1*B*).

Confirming previous results (Dutta et al., 2018), the nodal gap length in lifelong +dnVAMP2 mice maintained under normal visual conditions (12 h light/dark cycle) was enlarged compared with mice in which DOX was provided continuously (lifelong –dnVAMP2 mice) to maintain normal exocytosis from astrocytes. Mean optic nerve nodal gap lengths were significantly larger in lifelong +dnVAMP2 mice than lifelong –dnVAMP2 mice (Fig. 1*C*; lifelong –dnVAMP2 vs lifelong +dnVAMP2, respectively: mean nodal gap length ± SEM, 0.646 ± 0.026 vs 0.787 ± 0.036 μm; *t* test, *t*_(12)_ = −3.17, *p* = 0.008).

**Figure 2.**
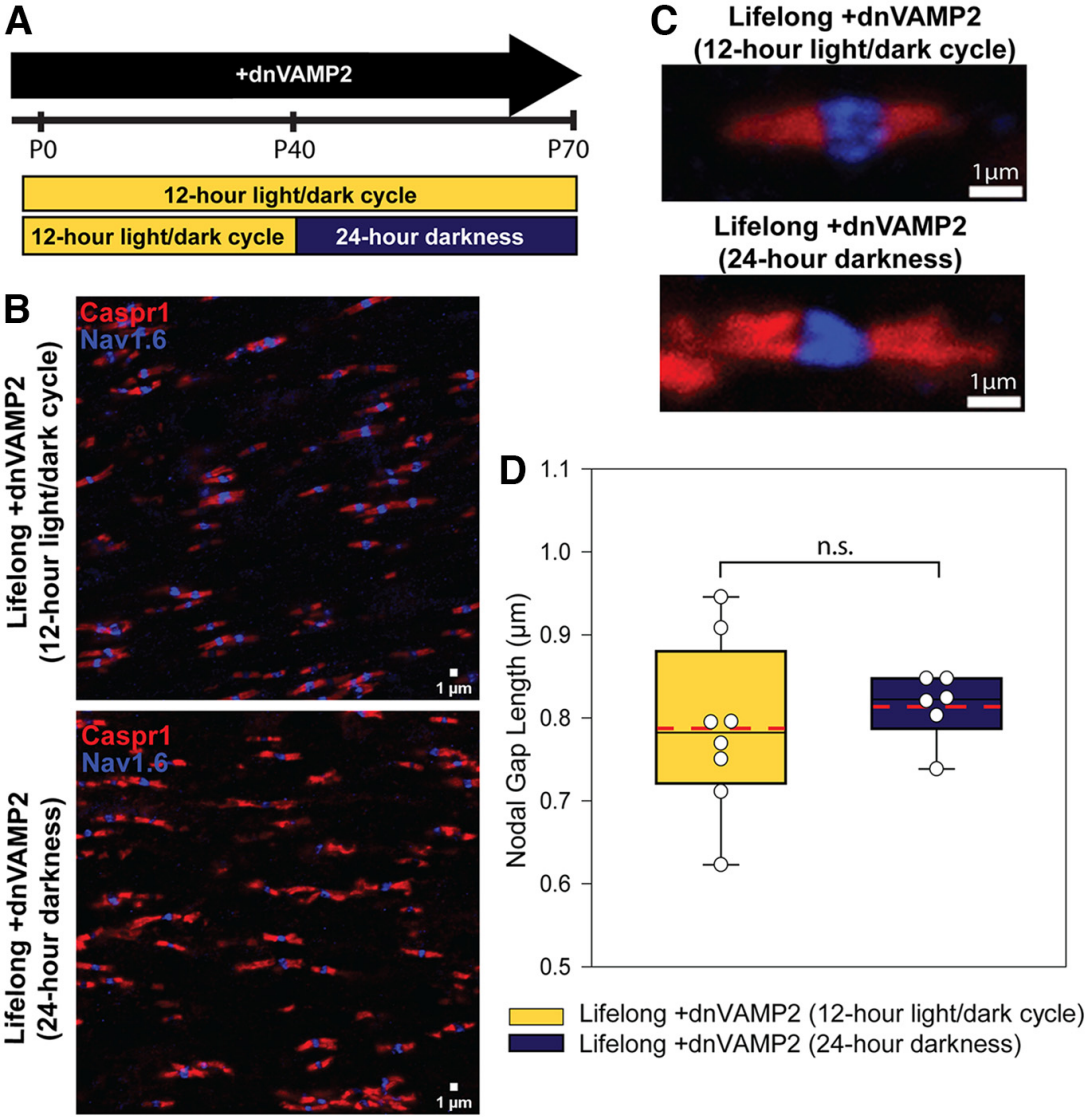
Astrocyte-regulated nodal lengthening proceeds normally during binocular visual deprivation. ***A***, Experimental timeline used to test whether visual experience influences the astrocyte-mediated mechanism of node of Ranvier plasticity. Exocytosis from astrocytes was reduced throughout life by withholding DOX from [gfap]dnVAMP2 transgenic mice throughout gestation and adulthood to express the dnVAMP2 gene continuously. At P40, after myelination of optic nerve is complete, a subset of these animals underwent 30 d of binocular deprivation (24 h darkness), and nodes of Ranvier were compared with similar animals experiencing normal visual conditions in a 12 h light/dark cycle. The lengths of the nodal gaps were compared in both groups of animals at P70. ***B***, Representative confocal microscope fields from +dnVAMP2 animals maintained under normal 12 h light/dark cycle (top) or under 24 h darkness (bottom) from P40 to P70. The paranodal region was labeled with Caspr1 (red), and the node was identified by sodium channel Na_v_1.6 immunocytochemistry (blue). ***C***, Examples of typical individual nodes of Ranvier from +dnVAMP2 animals maintained from P40 to P70 under the normal 12 h light/dark cycle (top) or under 24 h darkness (bottom). ***D***, Box plots of nodal gap length. Individual points represent the average of all nodes measured in each of 10 microscope fields per animal. Maximum, minimum, and median nodal gap lengths are represented within the box plot as whiskers (top and bottom) and mid-line (solid black). Mean nodal gap length of all animals within each condition is marked with a dashed red line. Nodal gap length was not significantly different in lifelong +dnVAMP2 animals following binocular visual deprivation compared with similar animals maintained under normal light conditions (mean nodal gap length ± SEM: 0.787 ± 0.036 vs 0.813 ± 0.017 μm; lifelong +dnVAMP2 12 h light/dark cycle versus lifelong +dnVAMP2 24 h darkness, respectively; *t* test, *t*_(9)_ = −0.65, *p* = 0.530). n.s. = not significant.

To determine whether sensory deprivation had an effect on this mechanism of nodal gap plasticity, adult mice were placed in complete darkness for 30 d, starting on P40, and nodes of Ranvier were examined by confocal microscopy at P70 (Fig. 2*A–C*). Nodal gaps were enlarged in both groups of animals maintained in darkness and under normal conditions when the dnVAMP2 gene was expressed. The enlarged nodal gap lengths were not statistically different comparing mice after BVD with mice maintained under normal light/dark conditions (Fig. 2*D*; mean nodal gap length ± SEM: 0.787 ± 0.036 vs 0.813 ± 0.017 μm; lifelong +dnVAMP2 12 h light/dark cycle vs lifelong +dnVAMP2 24 h darkness, respectively; *t* test, *t*_(9)_ = −0.65, *p* = 0.530). Thus, this mechanism of astrocyte-mediated lengthening of nodal gap length is not altered by 30 d of BVD in adult mice.

**Figure 3.**
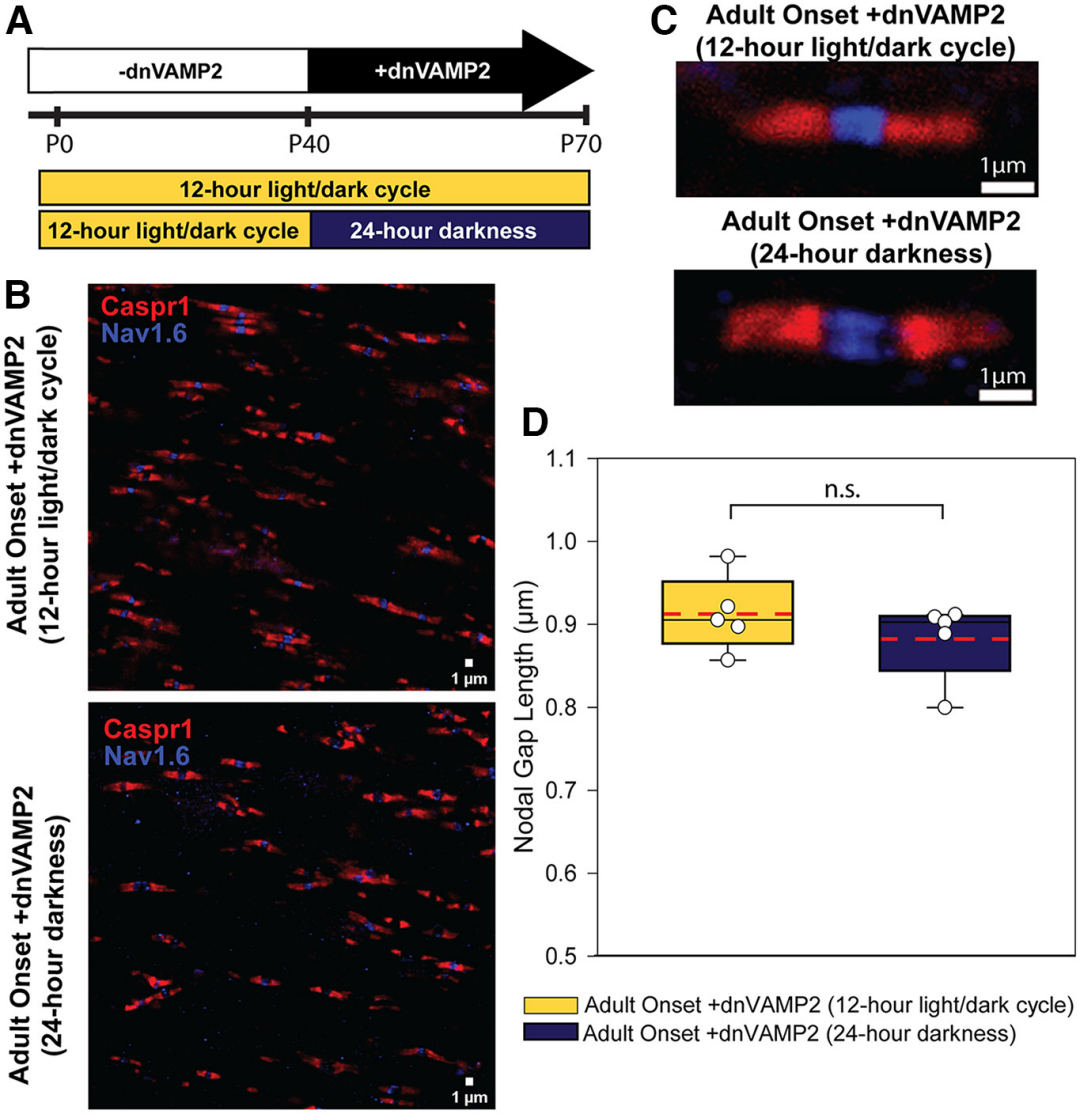
Reduction in astrocyte exocytosis in adult mice lengthened nodal gap length regardless of modulation of the visual system by dark exposure. ***A***, Experimental timeline for adult-onset +dnVAMP2 binocular deprivation used to eliminate possible confounds of reducing exocytosis in astrocytes in gestation and during myelination. Astrocyte vesicular release was reduced in adulthood via +dnVAMP2 expression begun at P40 by removing DOX from the diet at that time. The mice were then subjected to 30 d of binocular deprivation (24 h darkness) and nodes of Ranvier were compared at P70 with similar animals with normal visual experience (12 h light/dark cycle). ***B***, Representative confocal microscope fields from adult-onset +dnVAMP2 animals maintained under normal 12 h light/dark cycle (top) or under 24 h darkness (bottom) from P40 to P70. The paranodal region was labeled with Caspr1 (red), and the node was identified by sodium channel Na_v_1.6 (blue). ***C***, Examples of typical individual nodes of Ranvier from adult-onset +dnVAMP2 animals maintained from P40 to P70 under normal 12 h light/dark cycle (top) or under 24 h darkness (bottom). ***D***, Box plots of average nodal gap length measured per animal. Individual points represent the average of nodes of Ranvier in 10 microscope fields per animal. Maximum, minimum, and median nodal gap lengths a represented within the box plot as whiskers (top and bottom) and mid-line (solid black). Mean nodal gap length of all animals within each condition is marked with a dashed red line. Nodal gap length was not significantly different in adult-onset +dnVAMP2 animals following 30 d of binocular visual deprivation compared with similar animals maintained under normal light conditions (mean nodal gap length ± SEM: 0.912 ± 0.020 vs 0.882 ± 0.021 μm; adult-onset +dnVAMP2 12 h light/dark cycle vs adult-onset +dnVAMP2 24 h darkness, respectively; *t* test, *t*_(7)_ = 1.03, *p* = 0.336). n.s. = not significant.

Critical periods for experience-dependent plasticity in the visual system are well known, and inhibiting exocytosis from astrocytes throughout gestation could influence developmental events that could potentially impair responses to BVD experienced in adulthood. Therefore, exocytosis from astrocytes was left unperturbed during gestation by maintaining pregnant mice on a DOX diet throughout gestation and after weening offspring by supplying DOX to the diet of postnatal mice until P40 when myelination of optic nerve was complete. At P40, DOX was removed from the diet of these mice to induce adult expression of dnVAMP2 (Fig. 3*A*). Half of these adult-onset +dnVAMP2 mice underwent BVD for 30 d beginning on P40, and nodal gap lengths were compared at P70 with similar mice maintained under normal light/dark conditions. The results showed that reducing exocytosis throughout life, by expressing dnVAMP2 throughout gestation and early postnatal development, was not necessary for the increase in nodal gap length. Nodal gap was lengthened significantly when the reduction in astrocyte exocytosis was initiated in adulthood, by removing DOX from the diet at P40 to express the dnVAMP2 gene (mean nodal gap length ± SEM: 0.912 ± 0.020 vs 0.646 ± 0.026 μm; adult-onset +dnVAMP2 vs lifelong –dnVAMP2, respectively, both on a normal 12 h light/dark cycle; *t* test, *t*_(11)_ = −8.14, *p* < 0.001). BVD for 30 d, beginning on P40 in these adult-onset +dnVAMP2 mice, did not affect the process of nodal gap lengthening induced by inhibiting exocytosis from astrocytes in adulthood (Fig. 3*B–D*; mean nodal gap length ± SEM: 0.912 ± 0.020 vs 0.882 ± 0.021 μm; adult-onset +dnVAMP2 12 h light/dark cycle vs adult-onset +dnVAMP2 24 h darkness, respectively; *t* test, *t*_(7)_ = 1.03, *p* = 0.336).

Together these experiments indicate that nodal gap lengthening, after vesicle release from astrocytes is reduced either beginning in adulthood or maintained throughout life, proceeds similarly under conditions of prolonged BVD to when mice have normal visual experience.

### Effects of binocular visual deprivation on nodes of Ranvier in mice with normal vesicular release from astrocytes

**Figure 4.**
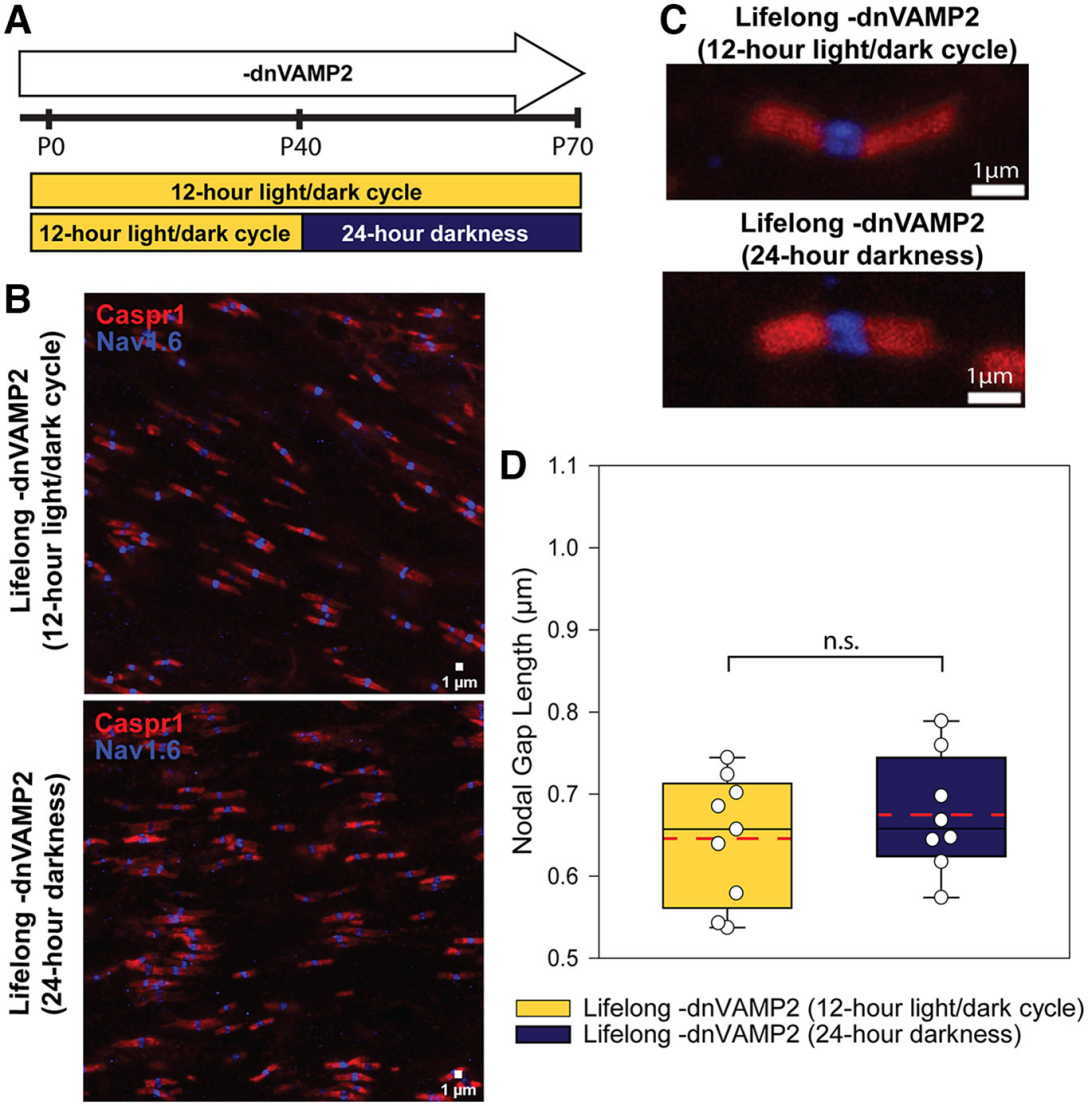
Long-term binocular deprivation does not alter length of nodes of Ranvier in adult optic nerves with normally functioning astrocyte vesicular release. ***A***, Experimental timeline to test whether binocular deprivation affects node of Ranvier gap length when exocytosis from astrocytes is unimpaired. Animals with unimpaired vesicular release from astrocytes were raised by supplying DOX during gestation and throughout life. A control group of lifelong –dnVAMP2 mice was maintained under normal visual conditions of a 12 h light/dark cycle from birth until P70, while similar animals undergoing binocular visual deprivation were raised under normal visual conditions of 12 h light/dark cycle until P40 and then maintained under 24 h darkness from P40 to P70. ***B***, Representative microscope fields from –dnVAMP2 animals maintained under normal 12 h light/dark cycle (top) or under 24 h darkness (bottom) from P40 to P70, the paranodal region, were labeled with Caspr1 (red), and the node is identified with Na_v_1.6 (blue). ***C***, Examples of typical individual nodes of Ranvier from –dnVAMP2 animals maintained under normal 12 h light/dark cycle (top) or under 24 h darkness (bottom) from P40 to P70. The paranodal region was labeled with Caspr1 (red), and the node was identified with sodium channel Na_v_1.6 immunostaining (blue). ***D***, Box plots of average nodal gap length measured per animal. Individual points represent the average of 10 microscope fields per animal. Maximum, minimum, and median nodal gap lengths are represented within the box plot as whiskers (top and bottom) and mid-line (solid black). Mean nodal gap length of all animals within each condition is marked with a dashed red line. Nodal gap length was not significantly different in lifelong –dnVAMP2 animals following binocular visual deprivation compared with similar animals maintained under normal light conditions (mean nodal gap length ± SEM: 0.646 ± 0.026 vs 0.675 ± 0.025 μm; lifelong –dnVAMP2 12 h light/dark cycle vs lifelong –dnVAMP2 24 h darkness, respectively; *t* test, *t*_(14)_ = −0.80, *p* = 0.435). n.s. = not significant.

Whether or not BVD induces nodal plasticity in mice under physiologically normal conditions is unknown. Therefore, the effects of BVD on nodal gap length in adult animals in which the [gfap]dnVAMP2 gene was not expressed (lifelong –dnVAMP2) were studied (Fig. 4). Confirming previous results, immunocytochemistry and immunoblotting confirmed that [gfap]dnVAMP2 mice maintained on DOX prenatally through adulthood have no expression of EGFP, indicating the successful suppression of the dnVAMP2 transgene (Fig. 1*B*,*C*), and thus have normal astrocytic function.

Mice with unimpaired astrocyte exocytosis (lifelong –dnVAMP2) were maintained under conditions that allowed lifelong normal visual experience and compared with mice after BVD in adulthood (Fig. 4). The results showed no significant difference in average nodal gap length in the –dnVAMP2 animals after 30 d of BVD compared with similar mice maintained under normal visual conditions (Fig. 4*D*; mean nodal gap length ± SEM: 0.646 ± 0.026 vs 0.675 ± 0.025 μm; lifelong –dnVAMP2 12 h light/dark cycle vs lifelong –dnVAMP2 24 h darkness, respectively; *t* test, *t*_(14)_ = −0.80, *p* = 0.435), indicating that BVD does not change nodal gap length in optic nerves of adult mice when mechanisms of nodal remodeling are unimpaired.

### Two-factor analysis of all experimental conditions

To perform a comprehensive analysis of sources of variance across all conditions in all experiments performed in this study, a two-way ANOVA using a general linear model fit was performed to determine the influence of vesicle release from astrocytes (DOX Treatment) and BVD (Visual Experience) on nodal gap length, and possible interaction between these two factors. In addition to the value of providing comparisons across all sample conditions, this analysis increases confidence in the results obtained using two-sample *t* tests.

**Figure 5.**
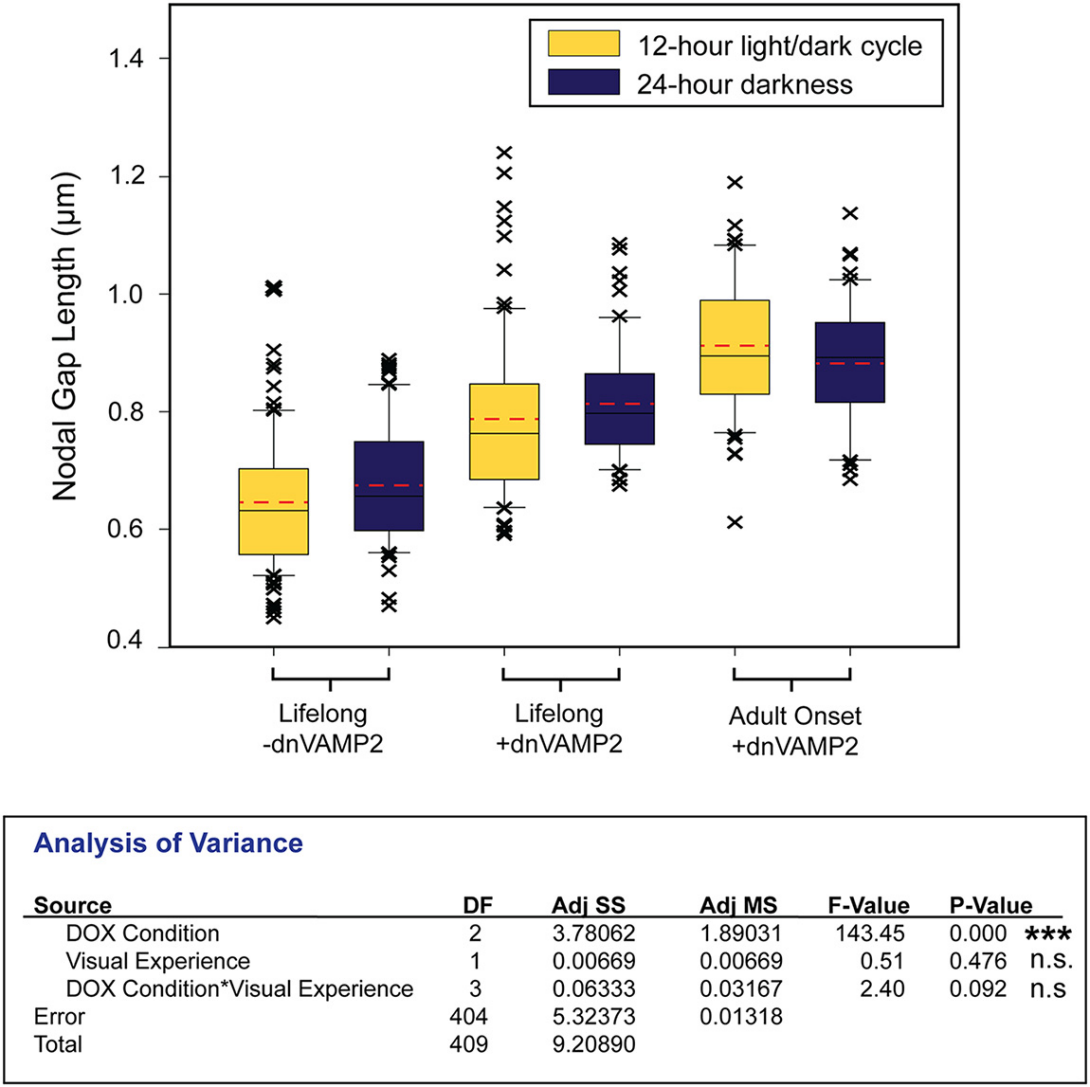
Two-way ANOVA of the effects of vesicle release from astrocytes and visual deprivation in all experimental conditions tested. The results show that vesicular release from astrocytes was the primary factor determining nodal gap length across all conditions (*p* < 0.001), and that sensory deprivation did not have a significant effect on nodal gap length (*p* = 0.476). There was no significant interaction between these two variables (*p* = 0.092; n.s. = not significant; *** *p* < 0.0001). Box plots of average nodal gap length measured per microscope field in all conditions. Outliers are represented with Xs. Maximum, minimum, and median nodal gap lengths are represented withing the box plots as whiskers (top and bottom) and midline (solid black). Mean nodal gap length of all animals within each condition is marked with a dashed red line.

An ANOVA, based on mean nodal gap lengths in each of 410 microscope fields from 41 mice, confirms the primary conclusions of this study (Fig. 5). Vesicular release from astrocytes (DOX treatment) was the main factor determining nodal gap length across all conditions, with a highly significant difference of (*p* < 0.001). BVD had no significant effect on nodal gap length (*p* = 0.476), and no interaction was found (*p* = 0.092).

## Discussion

The results showed that the astrocyte-mediated process of nodal gap lengthening was not altered significantly by maintaining adult mice in total darkness for 30 d. Whether reduction of astrocyte exocytosis was initiated in adult animals at the start of 30 d of BVD or was inhibited throughout life including during gestation, nodes of Ranvier were lengthened to a similar extent in mice experiencing BVD for 30 d as an adult as in mice having normal visual experience. This confirms and extends the previous finding that perinodal astrocytes have a predominant effect in regulating nodal gap length through exocytosis (Dutta et al., 2018), and that this mechanism proceeds despite prolonged BVD and in the presence of any other potential activity-dependent influences on nodal gap.

Furthermore, when exocytosis in astrocytes was unimpaired, BVD in darkness for 30 d begun in adult mice at P40 did not alter nodal gap length significantly. Therefore, the structure of the node of Ranvier is resilient to such marked changes in patterned neural activity produced by BVD. This could indicate the necessity of maintaining the reliability of action potential propagation over a wide range of activity levels, and that such plasticity may require different experimental paradigms. It has long been understood that monocular deprivation induces reorganization of the cortex such that afferents from the eye receiving patterned sensory information are favored (Wiesel and Hubel, 1965). If nodal length in optic nerve axons was increased by visual deprivation, which is a strong hypothesis considering new findings on myelin plasticity, longer latency of spike time arrival would, by spike time-dependent synaptic plasticity, decrease synaptic input from the deprived eye. Conversely, activity-dependent nodal gap length changes increasing conduction velocity would have the opposite effect. Our experimental results on optic nerve provide no support for this alternative hypothesis for the experience-dependent changes in synaptic strength reported in the visual system.

Across different experimental conditions, we used two-sample tests to directly assess the effect of BVD on animals of a similar genetic background. We took a conservative approach in using the number of animals as the sample size to minimize the probability of making a false-positive error that would be introduced by artificially increasing the sample size if the large number of individual nodes of Ranvier had been used as the sample size in statistical tests (24,747 nodes of Ranvier were measured in total). However, the more conservative approach that was taken necessarily increased the probability of a type II error of failing to detect a significant difference when there are differences (false negative); therefore, we also confirmed the primary conclusions of this study through a two-way ANOVA comparing the mean nodal gap length of nodes in 410 microscope fields measured across all conditions. This analysis confirmed that astrocyte exocytosis (regulated by DOX treatment) was the primary factor altering nodal gap length and that the visual experience condition had no significant effect or interaction.

Importantly, these findings do not exclude the possibility that nodal gap length may be regulated by visual experience under different experimental conditions. The pattern of action potential activity in optic nerve axons differs in the absence of visual input (Lowen et al., 2001), but visual deprivation does not block spontaneous action potentials. Spontaneous activity is present in the retina prenatally (Galli and Maffei, 1988) and postnatally (Meister et al., 1991). In contrast to BVD induced by total darkness, intraocular injection of TTX to block sodium-dependent action potentials does block activity-dependent synaptic plasticity in the visual cortex (Stryker and Harris, 1986). We explored the effects of intraocular injections of TTX in these experiments, but electrophysiological recordings of retinal function and visually evoked potentials in the visual cortex showed that TTX did not block neural activity for more than ∼24 h (data not shown), rendering the approach unsuitable for a 30 d activity block required for these experiments.

Although we find no effect of BVD on nodal gap length, previous studies have shown other myelination differences after manipulating visual experience during early postnatal development. A recent study indicates that long-term monocular deprivation shortens the length of the myelinated segments between the nodes of Ranvier in the optic nerve, increasing delays and the variability of spike time arrival (Etxeberria et al., 2016). Nodal gap length was not investigated in that study. Notably, the studies were conducted between P15 and P32, before the completion of myelin maturation, and the results were attributed to increased oligodendrogenesis, in contrast to our studies of myelin plasticity investigating adult myelin plasticity assayed at P70. Another study using *in viv*o two-photon imaging of layer 2/3 primary binocular visual cortex of adult mice shows that monocular deprivation has different effects on myelin plasticity in excitatory and inhibitory neurons (Yang et al., 2020). Remodeling of pre-existing myelin sheaths is observed on both types of axons under normal conditions, but monocular deprivation increases the incidence of myelin segments that either elongate or contract, causing movement of nodes of Ranvier along parvalbumin-expressing inhibitory neurons of the visual cortex, but not the projecting axons of excitatory neurons. These changes were accompanied by increases in axonal branch tip dynamics in inhibitory neurons, raising the question of whether myelin changes are secondary to neuronal plasticity. However, neither of these studies explores the effects of BVD or the changes in the structure of nodes of Ranvier.

It is possible that other experimental paradigms that have previously reported nodal remodeling may involve the astrocyte-mediated mechanism of nodal lengthening; however, not all reports of nodal lengthening align with the submicrometer-scale changes known to be regulated by the astrocyte. The large increase in nodal gap length in auditory nerve in response to noise-induced hearing loss differs from astrocyte-regulated mechanism of nodal plasticity, as it is characterized as an injury response that causes demyelination, according to the authors (Tagoe et al., 2014). Likewise, large increases in nodal gap length (several micrometers) are evident after blocking GABAergic signaling in oligodendrocyte progenitor cells, cells that can mature into oligodendrocytes (Benamer et al., 2020). Intriguingly, neurochemical excitotoxicity, induced by the application of glutamate, results in nodal expansion that resembles the astrocyte-mediated mechanism, as paranodal myelin loops were observed to lift from the axolemma and retract toward the internode; however, this pathologic hyperstimulation also resulted in nodal expansion on the order of several micrometers (Fu et al., 2009). In contrast to the large increases in nodal gap length accompanying pathologic or developmental effects, the magnitude of the nodal gap length increase observed after chronic sleep restriction is in the submicrometer range, similar to that produced by the astrocyte-mediated mechanism of nodal lengthening (de Vivo and Bellesi, 2019). Similarly, the magnitude of changes in nodal gap length reported after transcranial magnetic stimulation and spatial learning in adult mice (Cullen et al., 2021) is consistent with nodal plasticity regulated by the astrocyte, although those authors favor an unidentified process mediated by changes in axonal morphology.

Thus, many biological processes likely influence myelin and nodal structure, which may differ in the context of development, pathology, and activity-dependent plasticity. The lack of a difference in nodal remodeling under conditions of BVD by maintaining mice in darkness for 30 d, regardless of whether vesicular release from astrocytes was inhibited or remained normal, supports the hypothesis that the perinodal astrocyte is a primary intermediary in this form of plasticity.

In conclusion, our results, in the context of other research on myelin plasticity, support a process of remodeling nodes of Ranvier that is not produced by BVD. The possibility of nodal remodeling and myelin plasticity that is driven by an activity-dependent competitive process, rather than binocular deprivation, is an important area for future research.

## Synthesis

Reviewing Editor: Arianna Maffei, Stony Brook University Decisions are customarily a result of the Reviewing Editor and the peer reviewers coming together and discussing their recommendations until a consensus is reached. When revisions are invited, a fact-based synthesis statement explaining their decision and outlining what is needed to prepare a revision will be listed below. The following reviewer(s) agreed to reveal their identity: Stephen Van Hooser.

The reviewers agree that the negative results reported in the manuscript address an important question and provide a valuable contribution to the field. However, they both indicated, and I agree with them, that the manuscript lacks clarity and needs substantial rewriting to be considered for publication in eNeuro.

Additional concerns and requests for clarifications have been raised.

The detailed comments by the reviewers follow below.

Reviewer #1

The authors aimed at addressing whether nodal length modulation mediated by astrocytes is neuronal activity dependent, by using binocular visual deprivation (BVD) in adult mice. This is a very interesting question, which needs to be addressed to better understand activity dependent plasticity. However, the authors do not see any difference between control and BVD mice, suggesting neuronal activity is not implicated in nodal modulation in adult mice, or that the model used is not adapted. Ideally, the use of a second model of activity inhibition, or the use of a model where activity is increased, would make the study more compelling. Moreover, the rationale of further using the VAMP2 dominant negative model to assess whether “vesicular release from astrocytes could be one of the mechanisms for activity dependent plasticity” is not clear to me in the BVD model, where no difference in plasticity is observed. The intermingling of the two approaches (dominant negative line and BVD) further makes the logic of the manuscript very hard to follow.

A substantial rewriting of the manuscript is needed to clarify these issues.

Reviewer #2

The authors describe experiments that sought to examine whether dark-rearing produces changes in axonal plasticity at the node of Ranvier in the optic nerve, and whether any plasticity might be modulated by astrocytic expression of vescile-associated membrane protein 2 (VAMP2). The authors find that while dominant negative expression of VAMP2 produces a lengthening of nodal gap length, as in previous studies, there was no influence of dark-rearing on nodal gap length either in animals that had experienced dominant-negative VAMP2 throughout life or only in adulthood. It is a negative result, which is fine.

Major items:

1) The comparisons of prior knowledge of synaptic plasticity with the potential outcomes of the experiment are not parallel. The authors describe the changes in ocular dominance plasticity that are observed with monocular or binocular deprivation by lid suture, not dark-rearing. The site of many of the synaptic changes that the authors reference are at the thalamocortical synapse (Heynen .. Bear 2003) or within the cortex, but the authors study the optic nerve. Instead, the better studies for comparison are those that examine the retinogeniculate synapse directly (Chinfei Chen’s group comes to mind, e.g. Lin et al. 2014 and Hooks and Chen 2008). The idea that monocular deprivation by lid suture causes plasticity and that binocular deprivation by lid suture does not is not correct (binocular deprivation by lid suture wreaks havoc with orientation selectivity, although binocular deprivation by dark-rearing does not, White et al. 2001). The authors should discuss the parallel situation: binocular deprivation by dark-rearing is the manipulation and the optic nerve is the site of the measurement.

2) A negative result is fine and useful to publish, but I thought the manuscript had many more figures than necessary to communicate the results. I suggest moving the Supplementary Figure to be Figure 1 (one does have to consult it to be convinced that there is any effect of VAMP2). Then, use the motif of the current Figure 1 A to show all conditions, leave B, possibly expand C to show a couple more conditions, and then use the ANOVA graph and table from the last figure as D. Views of raw data from other conditions could go in Supplement. So, 2 paper figures total.

3) For a negative result, it is important to describe the biggest difference that one might have seen given the sample size. The authors can observe a change of about 0.15 microns in nodal gap length in Supplementary Figure D but is that at the limit? How big of a difference would the authors expect to be able to see with confidence?

4) There is no discussion about the change that one might predict in optic nerve conduction velocity or strength given a change from 0.65 micron gap length to 0.78 (or so) micron gap length. Would one notice? Is there any compatible reference?

Minor issues:

1) In the abstract, the authors should indicate that they use binocular deprivation by dark-rearing (there are many many forms of deprivation, it will help the reader to know in the abstract which was used here)

2) Fix the part in the abstract that makes comparisons to binocular physiological plasticity so it references a parallel situation (that is. Apply Major item 1 there)

3) Define OPCs

4) Apply Major item 1 to the last sentence in the introduction

5) Page 11, discussion (lines 346 - 351), apply Major item 1

6) Page 13, lines 416-420, apply Major item 1

## Author Response

Synthesis Statement for Author (Required):

The reviewers agree that the negative results reported in the manuscript address an important question and provide a valuable contribution to the field. However, they both indicated, and I agree with them, that the manuscript lacks clarity and needs substantial rewriting to be considered for publication in eNeuro.

Additional concerns and requests for clarifications have been raised.

The detailed comments by the reviewers follow below.

Reply: Thank you for these suggestions to improve the presentation of our results. The manuscript has been revised to address these suggestions.

In the original manuscript we complied with the 750-word limit for the Introduction, but this resulted in misunderstanding and confusion of the hypotheses being tested, because the restriction did not permit sufficient space to provide the necessary background information to understand why these studies were undertaken. To address this problem, identified by both reviewers and endorsed by the editor’s summary statement, we have moved the essential information that was in the opening of the Discussion into the Introduction. We feel this resolves the problem and is a necessary change. We ask that the journal permit an extension of the word limit in this case to address these fundamental issues that are essential to understand the study. Since this research bridges two traditionally separate fields (synaptic plasticity and myelin), there is a need to provide much more background information to understand the hypotheses, why, and how they are being tested, and to comply with reviewer requests to add specific new information.

We have added a reference to a recent paper published in BioRxiv (Pajevic et al., ), which provides a novel mechanism for how myelin plasticity can achieve optimal synchrony of spike time arrival using a different mechanism but parallel to STDP at synapses.

Reviewer #1

The authors aimed at addressing whether nodal length modulation mediated by astrocytes is neuronal activity dependent, by using binocular visual deprivation (BVD) in adult mice. This is a very interesting question, which needs to be addressed to better understand activity dependent plasticity. However, the authors do not see any difference between control and BVD mice, suggesting neuronal activity is not implicated in nodal modulation in adult mice, or that the model used is not adapted. Ideally, the use of a second model of activity inhibition, or the use of a model where activity is increased, would make the study more compelling. Moreover, the rationale of further using the VAMP2 dominant negative model to assess whether “vesicular release from astrocytes could be one of the mechanisms for activity dependent plasticity” is not clear to me in the BVD model, where no difference in plasticity is observed. The intermingling of the two approaches (dominant negative line and BVD) further makes the logic of the manuscript very hard to follow.

A substantial rewriting of the manuscript is needed to clarify these issues.

Reply: Thank you for your kind comments expressing appreciation of the importance of this research. We regret that the original manuscript allowed some apparent misunderstanding and confusion. To make the logic and significance of the studies clearer to readers, much of the pertinent information, which was originally at the opening of the Discussion, has been moved into the Introduction. We feel this improves the presentation, and we appreciate the reviewer bringing to light this possible point of confusion for readers. There is no confound between the two experiments. Each one addresses a different, independent hypothesis.

Briefly, the revisions clarify that two independent hypotheses are being tested, why the BVD model is appropriate, and explains the necessity of comparing our results in optic nerve with other research on synaptic plasticity in the visual system:

1. The recent discovery of activity-dependent myelin plasticity makes it necessary to revisit the classical experimental designs of Hubel and Wiesel (and others) to determine if activity-dependent changes in myelin, which affect spike time arrival, participate in the plasticity process they and others discovered. An implicit assumption in those studies was that conduction velocity in optic nerve was not altered by visual experience, but myelin plasticity could augment, inhibit, or even drive some of the outcomes of previous research on synaptic plasticity in the LGN and visual cortex performed before the recent discovery of myelin plasticity.

Our studies on mice that do not express the dnVAMPII gene, which would otherwise reduce vesicle release from astrocytes, show that binocular deprivation does not alter nodes of Ranvier in adult optic nerve, and this is an important finding. This result is in agreement with the established lack of changes in synaptic plasticity in LGN and visual cortex after BVD in adult animals. Therefore, results of other studies that will use or that have used BVD, are not complicated by this new form of plasticity. This finding will be of wide significance and critical to researchers using the BVD model to study visual system plasticity.

2. Regarding expanding the study to investigate alternative models of plasticity:

A number of alternative manipulations of neural activity could have been used, but BVD is the appropriate model to test the current hypothesis. Binocular deprivation is the fundamental model used to distinguish whether plasticity is driven by differences in the amount of neural activity or instead depends on more structured neural patterns of firing produced by sensory processing. Spontaneous action potential firing continues under BVD, but without the structured pattern of neural activity that is driven by visual experience or differences in activity between the two eyes. This important question has not been considered with respect to activity-dependent node of Ranvier plasticity. It is well established that BVD does not drive synaptic plasticity in the visual system, which is dependent upon activity-dependent competition between the two eyes. However, sensory deprivation could well alter node of Ranvier structure if it is a use/disuse phenomenon. Our results provide a clear answer. Nodes of Ranvier in optic nerve are not altered by BVD for 30 days in adult animals. This is an important finding, which has no relation to the other studies in our manuscript involving astrocyte-driven changes in nodes of Ranvier.

Results from different plasticity models may provide interesting comparisons, but undertaking different experimental models of plasticity would expand well beyond the hypothesis being tested and they are not necessary to test this hypothesis regarding whether sensory deprivation affects nodal structure. The findings from our BDV experiments will provide the basis for research using different experimental models by others (and ourselves) in the future.

3. Regarding dnVAMPII experiments:

A different, but very important question, concerns the recently discovered mechanism of node of Ranvier and myelin thickness plasticity in adult animals that is regulated by manipulating vesicle release of thrombin inhibitors from astrocytes. It is not known whether functional activity affects this process. This is an important question because other studies of activity-dependent nodal gap lengthening are observational, without a specific mechanism for the plasticity in mature myelin. To advance the field, it is essential to begin to investigate specific mechanisms of node of Ranvier plasticity and determine if they are influenced by action potential activity.

There is no contradiction between our studies in dnVAMPII mice and our experiments in mice not expressing this gene, which is used to investigate a different hypothesis (above). It is important to emphasize that the plasticity in dnVAMII mice is driven by the transgene, not by BVD. For this reason, BVD in dnVAMPII expressing mice is used as the means to investigate if sensory experience influences this specific mechanism of nodal plasticity.

By analogy to synaptic plasticity research, expression of a specific protein could be altered, reducing synaptic transmission, and then the question of whether functional activity influences the outcome could be determined by BVD studies after that molecular intervention. Such studies are common in the field of synaptic plasticity.

Our results show that BVD does not alter the effects of dnVAMPII expression in astrocytes on lengthening the nodal gap. This is a compelling hypothesis to test, because any number of signaling molecules released by axons firing action potentials could influence astrocytes, oligodendrocytes, or axons at the node to change nodal gap width. That hypothesis is rejected by the results providing no evidence for these alternatives.

Reviewer #2

The authors describe experiments that sought to examine whether dark-rearing produces changes in axonal plasticity at the node of Ranvier in the optic nerve, and whether any plasticity might be modulated by astrocytic expression of vescile-associated membrane protein 2 (VAMP2). The authors find that while dominant negative expression of VAMP2 produces a lengthening of nodal gap length, as in previous studies, there was no influence of dark-rearing on nodal gap length either in animals that had experienced dominant-negative VAMP2 throughout life or only in adulthood. It is a negative result, which is fine.

Reply: Thank you for this summary and for endorsing the importance of publishing results of this nature. It is important, however, to clarify that dark-rearing is not used in our studies. All of our studies are on visual deprivation in adult animals.

Major items:

1) The comparisons of prior knowledge of synaptic plasticity with the potential outcomes of the experiment are not parallel. The authors describe the changes in ocular dominance plasticity that are observed with monocular or binocular deprivation by lid suture, not dark-rearing. The site of many of the synaptic changes that the authors reference are at the thalamocortical synapse (Heynen .. Bear 2003) or within the cortex, but the authors study the optic nerve. Instead, the better studies for comparison are those that examine the retinogeniculate synapse directly (Chinfei Chen’s group comes to mind, e.g. Lin et al. 2014 and Hooks and Chen 2008). The idea that monocular deprivation by lid suture causes plasticity and that binocular deprivation by lid suture does not is not correct (binocular deprivation by lid suture wreaks havoc with orientation selectivity, although binocular deprivation by dark-rearing does not, White et al. 2001). The authors should discuss the parallel situation: binocular deprivation by dark-rearing is the manipulation and the optic nerve is the site of the measurement.

Reply: Cross comparisons of our results in optic nerve with effects of sensory deprivation on LGN and visual cortex are appropriate because changes in conduction velocity from node of Ranvier remodeling in optic nerve will alter spike time arrival at these down-stream points and influence STDP.

We have revised this text to more carefully describe studies in visual system plasticity using different experimental approaches, and we added the requested references. This material was presented in the Discussion in the original text, but the presentation is improved by doing so earlier in the Introduction.

It is important to emphasize that are studies do not use dark-rearing. All our studies are on adult animals subjected to 30 days BVD, performed after myelin is fully formed and later than the periods in the papers by Lin 2014, and Hooks and Chen 2008, studying synaptic plasticity and maturation in LGN, which are now cited.

The following text has been inserted:

"The effects of BVD on synaptic plasticity, and other cellular/biochemical responses, depend on the experimental parameters used; notably, when in postnatal development sensory deprivation is imposed and how BVD is produced. The effects of BDV on synaptic development and maturation can differ in cortical and subcortical regions, with age, the timing and length of sensory deprivation, species, and whether deprivation is produced by darkness, eyelid suture, or pharmacology (Hooks et al., 2008; Liu et al., 2015; White et al., 2001).”

2) A negative result is fine and useful to publish, but I thought the manuscript had many more figures than necessary to communicate the results. I suggest moving the Supplementary Figure to be Figure 1 (one does have to consult it to be convinced that there is any effect of VAMP2). Then, use the motif of the current Figure 1 A to show all conditions, leave B, possibly expand C to show a couple more conditions, and then use the ANOVA graph and table from the last figure as D. Views of raw data from other conditions could go in Supplement. So, 2 paper figures total.

Reply: We are gratified by the acceptance of our results and endorsing their importance for publication. We carefully considered the suggestions for re-grouping and condensing information in the figures.

As requested, we have moved Supplemental Figure 1 into the main text as Figure 1.

Thank you for the suggestion of combining multiple figures into one, but we feel that using separate figures to provide the results from each of the different experiments separately conveys the results most clearly. The experimental details are complex, and the apparent confusion regarding the different hypotheses tested and different experimental designs used would likely increase if figures were combined. Also, we are reluctant to make such large changes in the figures unilaterally when the other reviewer did not express a concern with the figures.

3) For a negative result, it is important to describe the biggest difference that one might have seen given the sample size. The authors can observe a change of about 0.15 microns in nodal gap length in Supplementary Figure D but is that at the limit? How big of a difference would the authors expect to be able to see with confidence?

Reply: Possibly we are misunderstanding the question. The p value for these data is < 0.008, indicating a very high level of confidence in the mean differences. We provide all the statistical results in the manuscript in standard APA format, including the t-statistic value, SEM, and df, for every result in the paper, which enables readers to make further statistical calculations, if desired, such as calculating the minimal detectable difference at any probability level. It seems that the reviewer may be asking what is the smallest difference that could be detected at the p < 0.05 level, given the df, and variances for these data. If so, this can be calculated from the statistical results provided, which is 0.078 um. This is much smaller than the highly significant 0.15 um change in nodal gap length reported.

4) There is no discussion about the change that one might predict in optic nerve conduction velocity or strength given a change from 0.65 micron gap length to 0.78 (or so) micron gap length. Would one notice? Is there any compatible reference?

Reply: Thank you for this suggestion. This is good information include, and we have added it to the text. We reported previously (Dutta et al., 2018), that from electrophysiological measurements of conduction velocity in optic nerve, and consistent with mathematical modeling from axon cable properties, that this change in nodal gap width caused by reducing exocytosis in astrocytes in dnVAMPII expressing mice, results in approximately 20% slowing of CV in optic nerve. Our recordings in visual cortex show that this results in a 6.7 ms increase in latency of spike time arrival in visual cortex, which is substantial given the 72.8 ms latency to peak visually evoked potential. Optomotor measurements of visual acuity indicate a decrease in visual acuity of 0.01 cpd (cycles per degree). All of these differences are statistically significant.

Minor issues:

1) In the abstract, the authors should indicate that they use binocular deprivation by dark-rearing (there are many many forms of deprivation, it will help the reader to know in the abstract which was used here)

Reply: Dark-rearing was not used in our experiments. BVD was used in adult animals. We have added “by maintaining adult animals in the dark for 30 days” to clarify the BVD treatment used and to avoid this misunderstanding.

2) Fix the part in the abstract that makes comparisons to binocular physiological plasticity so it references a parallel situation (that is. Apply Major item 1 there)

Reply: We feel that this comparison is important to make in the abstract, which should present the wider significance of the findings. A significant outcome of our results is how our findings on nodal plasticity in optic nerve axons relate to previous studies of BVD performed throughout the visual system at molecular, cellular, and electrophysiological levels. These prior studies were done before activity-dependent plasticity of nodes of Ranvier was known, so they were performed and interpreted under the assumption that conduction velocity in the optic nerve was not altered by BVD. Our findings validate that untested assumption. Had BVD changed nodal structure and conduction velocity in optic nerve, this could have counteracted, reinforced, or even driven the changes reported through the visual system where spike time arrival is paramount.

3) Define OPCs

Reply: Done.

4) Apply Major item 1 to the last sentence in the introduction

Reply: The Introduction has been revised to address this point and the suggestions of the other reviewer.

5) Page 11, discussion (lines 346 - 351), apply Major item 1

Reply: Done.

6) Page 13, lines 416-420, apply Major item 1

Reply: The point is well taken, and this matter is now addressed by changes to the Introduction and by the additional references, as explained in our response to Major item 1 above, but these lines at the end of the Discussion reference the two specific papers by Hubel and Wiesel, which are cited. Expanding beyond them and restating the information on the effects of other experimental approaches would not be appropriate here. Also, we hope the revisions to the text make clear that neither eyelid suture nor dark rearing were used in our experiments.
